# Modulation of Amyloid-β and Tau in Alzheimer’s Disease Plasma Neuronal-Derived Extracellular Vesicles by Cerebrolysin® and Donepezil

**DOI:** 10.3233/JAD-220575

**Published:** 2022-11-08

**Authors:** X. Anton Alvarez, Charisse N. Winston, James W. Barlow, Floyd M. Sarsoza, Irene Alvarez, Manuel Aleixandre, Carlos Linares, Manuel García-Fantini, Birgit Kastberger, Stefan Winter, Robert A. Rissman

**Affiliations:** a Medinova Institute of Neurosciences, Clinica Reha Salud, A Coruña, Spain; b Clinical Research Department, QPS Holdings, A Coruña, Spain; c Department of Neurosciences, University of California, San Diego, CA, USA; d School of Psychology, Granada University, Granada, Spain; e Complejo Asistencial HHSCJ, Málaga, Spain; f Hospital HM Modelo, A Coruña, Spain; g EVER Neuro Pharma, Unterach, Austria; h VA San Diego Healthcare System, San Diego, CA, USA

**Keywords:** Aβ_42_, Alzheimer disease, Cerebrolysin®, combination therapy, donepezil, plasma neuronal-derived extracellular vesicles, tau

## Abstract

**Background:**

Plasma neuronal-derived extracellular vesicles (NDEV) contain proteins of pathological, diagnostic, and therapeutic relevance.

**Objective:**

We investigated the associations of six plasma NDEV markers with Alzheimer’s disease (AD) severity, cognition and functioning, and changes in these biomarkers after Cerebrolysin®, donepezil, and a combination therapy in AD.

**Methods:**

Plasma NDEV levels of Aβ_42_, total tau, P-T181-tau, P-S393-tau, neurogranin, and REST were determined in: 1) 116 mild to advanced AD patients and in 20 control subjects; 2) 110 AD patients treated with Cerebrolysin®, donepezil, or combination therapy in a randomized clinical trial (RCT). Samples for NDEV determinations were obtained at baseline in the NDEV study and at baseline and study endpoint in the RCT. Cognition and functioning were assessed at the same time points.

**Results:**

NDEV levels of Aβ_42_, total tau, P-T181-tau, and P-S393-tau were higher and those of neurogranin and REST were lower in mild-to-moderate AD than in controls (*p* < 0.05 to *p* < 0.001). NDEV total tau, neurogranin, and REST increased with AD severity (*p* < 0.05 to *p* < 0.001). NDEV Aβ_42_ and P-T181-tau correlated negatively with serum BDNF (*p* < 0.05), and total-tau levels were associated to plasma TNF-α (*p* < 0.01) and cognitive impairment (*p* < 0.05). Combination therapy reduced NDEV Aβ_42_ with respect to monotherapies (*p* < 0.05); and NDEV total tau, P-T181-tau, and P-S396-tau were decreased in Cerebrolysin-treated patients compared to those on donepezil monotherapy (*p* < 0.05).

**Conclusion:**

The present results demonstrate the utility of NDEV determinations of pathologic and synaptic proteins as effective AD biomarkers, as markers of AD severity, and as potential tools for monitoring the effects of anti-AD drugs.

## INTRODUCTION

Amyloid-β (Aβ), tau pathology, and the loss of synaptic elements are early neuropathological events and useful biomarkers in the Alzheimer’s disease (AD) continuum [1–5]. AD is characterized by the loss of synapses and the pathologic accumulation of Aβ and hyperphosphorylated tau into the brain [4, 5]; as well as by reduced levels of Aβ and synaptic proteins, and enhanced concentrations of tau and phosphorylated tau in the cerebrospinal fluid (CSF) [1–3]. The detection of these brain and CSF biomarkers involves complicated, expensive and/or invasive procedures that limit their routine use; and, therefore, the implementation of reliable peripheral biomarkers is a priority in current AD research [6, 7]. Neuronal-derived extracellular vesicles (NDEV) are extracellular microvesicles of endosomal origin released by neurons of the central nervous system that are able to cross the blood-brain barrier and contain biologically active cargo of tissue-specific molecules which can be detected in the peripheral blood [7–10]. Recent investigations in AD demonstrated that circulating NDEV contain pathological molecules such as Aβ_142_ (Aβ_42_), total and phosphorylated tau, and synaptic proteins [10–12]; which may contribute to the spread of AD pathology [9, 13, 14], and can serve as effective disease biomarkers [7–9, 15, 16].

In AD patients, plasma NDEV levels of Aβ_42_ were found to be increased as compared with controls [10–12, 17–20], to correlate with CSF Aβ_42_ levels and with PET imaging of brain amyloid plaque load [12, 19], but not with measures of cognitive impairment [18], and to represent an efficient blood biomarker [7]. Enhanced plasma NDEV levels of total tau, tau phosphorylated at threonine 181 (P-T181-tau), and tau phosphorylated at serine 396 (P-S396-tau) have also been reported in AD cases with respect to controls [10–12, 17, 21], but some authors failed to show such group differences for several of these tau parameters [11, 22, 23]. NDEV total tau and P-T181-tau correlated positively with their CSF values [12]; and higher plasma levels of total tau were found to be associated with lower cognitive performance [22] and with greater cognitive decline in AD [21]. Neurogranin (NRGN) is a post-synaptic protein that has been involved in the regulation of synaptic plasticity and long-term potentiation through calcium-mediated mechanisms [16]. NRGN expression was reported to be reduced in AD parietal and temporal cortices. Moreover, reduced NRGN expression was shown to be associated with the degree of Aβ and tau pathology [24]; although other authors found no differences in the levels of NRGN in Brodmann brain areas BA22 (in the superior temporal gyrus) and BA6 (in the frontal cortex) between AD and controls [25]. Several studies showed that NRGN levels were elevated in the CSF [16] and decreased in plasma NDEV [10, 16–18, 26] of AD patients. Together these data support the use of CSF and NDEV NRGN as a cognitive biomarker in AD [16]. Repressor element 1-silencing transcription factor (REST), also called neuron-restrictive silencing factor, regulates genes mediating cell death, stress resistance, and AD pathology; is induced in the aging human brain and almost absent in the nucleus of vulnerable cortical and hippocampal neurons of AD brains; and correlates with cognitive preservation during aging and with longevity [27]. Reduced levels of REST in plasma [28] and plasma NDEVs [10, 17, 29], but not in serum [30] have been demonstrated and represent a candidate biomarker in AD. However, changes in these plasma NDEV markers with disease severity and their correlations with measures of cognitive performance have been poorly investigated in AD. Here, in this study we measured plasma NDEV levels of Aβ_42_, total tau, P-T181-tau, P-S396-tau, NRGN, and REST in 116 patients with mild to moderately severe AD and in 20 cognitively normal control subjects. We then analyzed changes in the levels of these blood biomarkers as a function of AD severity; and assessed their correlations with cognition and functioning scores, and with serum brain-derived neurotrophic factor (BDNF) and plasma tumor necrosis factor-α (TNF-α) concentrations.

Brain derived extracellular vesicles (BEVs) are also potential therapeutic tools for AD [8, 31], and blood plasma NDEV biomarkers could be useful for monitoring the effects of anti-AD drugs [32]. Finally, we evaluated changes in the levels of the aforementioned NDEV parameters induced by the treatment with Cerebrolysin®, donepezil or a combination of both drugs in AD patients included in a randomized clinical trial [33]. Cerebrolysin® is a peptidergic preparation showing clinical efficacy in AD [2, 33, 34], and the ability of reducing Aβ [35–39], tau [40–42], and synaptic pathology [35, 36] in several experimental models. Donepezil is a cholinesterase inhibitor approved for the symptomatic treatment of AD that was reported to reduce Aβ levels *in vitro*, in the brain of a transgenic model of AD and in the serum of AD patients [43–46], and to increase the levels of tau protein in SH-SY5Y cells [47].

## METHODS

### Study design and participants

In this study, two sets of participants’ samples were included and processed for neuronal EV cargo. First, in an NDEV Study we determined the levels of Aβ_42_, total tau, P-T181-tau, P-S396-tau, NRGN, and REST in plasma neuronal derived cellular vesicles obtained from 116 patients (93 females) with mild to moderately severe AD (39 mild, 42 moderate, and 35 advanced AD cases) and from 20 (15 females) healthy elderly controls. All participants were recruited and evaluated through the Memor@ctiva Program of the Medinova Institute of Neurosciences at Clinica RehaSalud (A Coruña, Spain), from January 2014 to July 2017. Diagnosis of probable AD was done according to NINCDS-ADRDA [48] and DSM-V [49] criteria; and patients with Mini-Mental State Examination (MMSE) scores between 12 and 25 were selected. Elderly controls with a MMSE score higher than 25 were included. We also assessed the influence of treatment on the levels of plasma NDEV biomarkers in samples available from 110 patients with mild to moderate probable AD who completed a randomized clinical trial (RCT) (clinicaltrials.gov number NCT00911807) according to the protocol. Eligible RCT patients had MMSE scores of 12–25 and were receiving double-dummy treatment with Cerebrolysin® (10 mL, five IV infusions/week at weeks 1–4 and 13–16, *n* = 38), donepezil (5 mg once daily at weeks 1–4 and 10 mg once daily at weeks 5–28, *n* = 36) or combination therapy of both (*n* = 36) as previously described [33, 50].

Subjects having any other significant neurological or psychiatric disease, active allergies, unstable medical conditions, or clinically significant laboratory abnormalities were excluded. Brain CT and/or MRI examinations supporting the clinical diagnosis were required. Apolipoprotein E (*APOE*) genotype was available for AD patients and control cases. Patients and controls were not taking systemic corticosteroids, anti-parkinsonian agents, narcotics, or cholinesterase inhibitors for at least one month prior to blood sampling. Patients showing clinically significant depression in the medical evaluation and/or scores higher than fifteen points in the 17-item subscale of the Hamilton Depression Scale [51] were not included in the study. None of the participants reported changes in the general level of physical activity in the month prior study evaluations. The study was conducted in accordance with the last version of the Declaration of Helsinki, with Good Clinical Practice guidelines, and with applicable regulatory requirements. Written informed consent was obtained from all participants before starting study procedures. Study protocol was approved by an independent ethics committee. Patient data was processed in compliance with the regulations on personal data protection.

### Measurements of L1CAM-positive NDEV cargo proteins

Blood samples for determinations of NDEV proteins were obtained at baseline for AD and control cases; as well as at baseline and week 28 (study endpoint) for RCT AD patients. Isolation and characterization of L1CAM-positive NDEV from plasma were carried out according to procedures previously described [52]. Briefly, EVs were extracted and precipitated from 250μL of human plasma, using established methodology as previously described [52]. Extracted EVs were enriched against a neuronal protein (L1CAM; eBio5G3, Biotin, eBioscience) using magnetic immunocapture (EXOFLOW; System Biosciences, Inc.) and fluorescence-activated cell sorting (FACS) sorting (BD FACS Aria II) [52]. Nanoparticle Tracking Analysis (NTA) was used to characterize NDEVs based on size distribution [10]. Protein concentrations for NDEVs preparations were determined using a bicinchoninic acid (BCA) Protein Assay kit (Pierce Biotechnology). L1CAM-positive NDEV cargo proteins were quantified by human-specific ELISAs for P-T181-tau (Fujirebio US, Inc., Alpharetta, GA), Aβ_42_, P-S396-tau, and total tau (Life Technologies/Invitrogen, Camarillo, CA), neurogranin (Cloud Clone Corp, American Research Products-Katy, TX), REST and tetraspanning EV marker CD81 (Cusabio, American Research Products–Waltham, MA) according to suppliers’ directions. The mean value for all determinations of CD81 in each assay group was set at 1.00, and the relative values for each sample were used to normalize their recovery. Evidence for enrichment of exosomes from neural sources in plasma has been demonstrated previously [52, 53].

### Clinical and other laboratory evaluations

Cognitive performance was evaluated by using the MMSE [54] and the Alzheimer’s Disease Assessment Scale-cognitive subscale modified (ADAS-cog+) [55], a 14-item extended version of the AD Assessment Scale-cognitive subscale with an increased sensitivity to detect cognitive changes in milder patients. Functioning was assessed with the AD Cooperative Study-Activities of Daily Living Scale (ADCS-ADL) [56]. AD severity was graded on the 7-point Clinical Interview Based Impression of Severity with Caregiver Input scale (CIBIS+) and patients CIBIS+scores 3–5 were included (3 = mild AD; 4 = moderate AD; 5 = advanced AD) [57]. In the ADAS-cog+, a lower score/negative score of change indicates a better cognitive performance/cognitive improvement; and the opposite applies for ADCS-ADL functioning. Age, sex, *APOE* genotype, platelet counts, BDNF, and TNF-α were also considered for data analysis.

### Statistical analysis

**Fig. 1:**
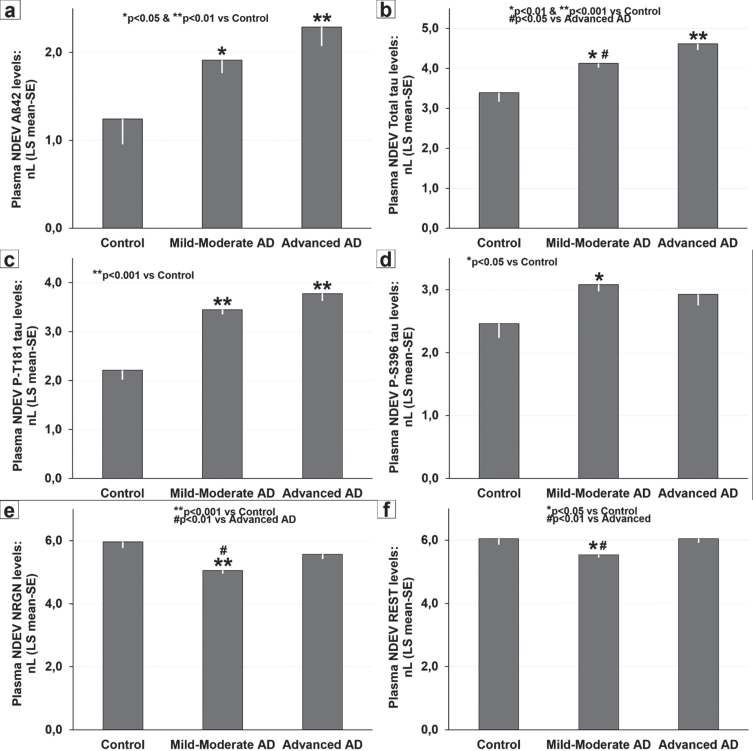
Severity-related changes in baseline plasma neuronal-derived extracellular vesicles (NDEV) levels of: (a) Aβ_42_, (b) total-tau, (c) P-T181-tau, (d) P-S396-tau, (e) NRGN, and (f) REST in controls, mild-to-moderate AD, and advanced AD. (a) *p < 0.05 and **p < 0.01 versus controls; (b) *p < 0.01 and **p < 0.001 versus controls, and #*p* < 0.05 versus advanced AD; (c) **p < 0.001 versus controls; (d) *p < 0.05 versus controls; (e) **p < 0.001 versus controls, and #*p* < 0.01 versus advanced AD; (f) *p < 0.05 versus controls, and #*p* < 0.01 versus advanced AD. Data are presented as LS mean (±SE) of natural log (nL) values and were analyzed by ANCOVA.

**Fig. 2:**
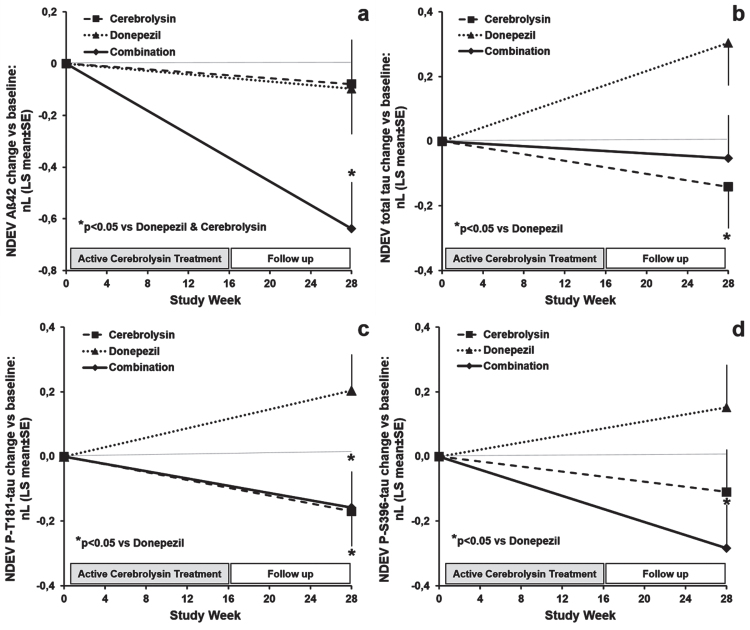
Effects of Cerebrolysin®, donepezil, and the combination therapy on plasma neuronal-derived extracellular vesicles (NDEV) levels of: (a) Aβ_1–42_, (b) total-tau, (c) P-T181-tau, and (d) P-S396-tau at week 28 (end of the study) in patients with mild-to-moderate AD. (a) *p < 0.05 versus donepezil and Cerebrolysin® groups; (b, c, d) *p < 0.05 versus donepezil group. Data are presented as LS mean (±SE) change from baseline of natural log (nL) values and were analyzed by ANCOVA.

NDEV Aβ_42_, total-tau, P-T181-tau, P-S396-tau, NRGN, and REST levels were analyzed in relation to CD81 values. CD81-adjusted data for these parameters did not follow a normal distribution. In order to allow the control of non-normality and the influence of covariates, NDEV concentrations of biomarkers were log-transformed and parametric statistics were used for the analysis of natural log (nL) data. Group comparisons were done by Chi-Square, ANOVA, and ANCOVA analyses as appropriate. Since plasma NDEV levels were similar in mild and moderate AD cases for all the six biomarkers investigated, these two AD stages were merged for severity-related statistical analyses. NDEV biomarkers data are presented as nL means plus/minus standard deviations (X (SD)) in Tables 1-2 and as estimated nL means plus/minus standard errors (X (SE)) in Fig. 1; and CD81-adjusted scores are also described in the results section. Treatment differences in the changes of NDEV biomarkers from baseline to week-28 were analyzed by ANCOVA using nL data scores of change from baseline as dependent variable and baseline nL values as covariate with appropriate corrections for age, gender, platelet counts, disease severity, and *APOE4* status (*APOE* ɛ4 allele present or not). Estimated least square means change plus/minus standard error (LS mean (SE)) of NDEV biomarkers are indicated for each treatment group in Table 3 and Fig. 2. Partial correlation analysis with appropriate corrections for age, gender, *APOE4* status, and disease severity was employed. Probability values lower than 0.05 were considered statistically significant.

**Table 1:** Plasma neuronal-derived extracellular vesicles (NDEV) biomarkers in AD patients and controls

	Control Group (*N* = 20)	AD Group (*n* = 116)	Analysis		
	*N (%)*	*N (%)*	χ*^2^*	*df*	*p*
Females	15 (75.0)	93 (80.2)	0.279	1	0.597
	*Mean*±*SD*	*Mean*±*SD*	*F*	*df*	*p*
Age (y)	74.35±5.76	74.87±7.55	0.086	1, 134	0.770
MMSE (score)	28.10±0.91	17.59±4.70	98.321	1, 134	**0.000**
ADAS-cog+(score)	17.07±4.71	41.10±17.54	24.024	1, 127	**0.000**
Plasma NDE levels (nL values)	*Mean* ± *SD*	*F*	*df*	*p*
Aβ_42_	1.26±0.91	2.02±1.37	5.634	1, 131	**0.019**
Total-tau	3.39±0.88	4.26±0.98	11.102	1, 129	**0.001**
P-T181-tau	2.35±1.40	3.54±0.83	36.592	1, 134	**0.000**
P-S396-tau	2.64±1.46	3.03±0.93	5.217	1, 130	**0.024**
NRGN	5.78±0.88	5.20±0.93	12.104	1, 133	**0.001**
REST	6.08±0.58	5.69±0.89	3.152	1, 130	**0.078**

Aβ_42_, amyloid-β 1-42; ADAS-cog+, AD assessment scale-cognitive subscale modified; MMSE, Mini-Mental State Examination; nL, natural log; NRGN, Neurogranin; P-T181-tau, tau phosphorylated at threonine 181; P-S396-tau, tau phosphorylated at serine 396; REST, repressor element 1-silencing transcription factor. Chi-Square (χ^2^) and ANOVA (F) analyses were employed.

**Table 2:** Effects of Cerebrolysin®, donepezil, and combination therapy on plasma neuronal-derived extracellular vesicles (NDEV) biomarkers in AD: Baseline clinical data and week 28 results

	Cerebrolysin®	Donepezil	Combination	Analysis		
	(*n* = 38)	(*N* = 36)	(*n* = 36)
	*N (%)*	*N (%)*	*N (%)*	χ*^2^*	*df*	*p*
Females	31 (81.6)	28 (77.8)	31 (86.1)	0.843	2	0.656
*APOE* ɛ4 allele	19 (50.9)	15 (41.7)	15 (41.7)	0.699	2	0.705
	*Mean*±*SD*	*Mean*±*SD*	*Mean*±*SD*	*F*	*df*	*p*
Age (y)	75.44±6.21	76.08±7.87	72.52±8.12	2.355	2, 107	0.100
MMSE (score)	17.26±4.62	17.69±4.52	17.63±5.03	0.092	2, 107	0.913
ADAS-Cog+(score)	41.38±16.11	40.80±17.56	40.73±19.75	0.015	2, 107	0.985
Plasma NDE levels (nL)	*Mean* ± *SD*			*F*	*df*	*p*
Baseline Aβ_42_	1.74±1.36	1.87±1.63	1.68±1.21	0.170	2	0.844
Week-28 Aβ_42_	1.65±1.36	1.74±1.53	1.08±1.54	2.040	2	0.135
Baseline Total-tau	4.12±0.93	4.21±0.97	4.18±1.18	0.077	2	0.926
Week-28 Total-tau	4.01±0.95	4.51±0.92	4.06±1.01	2.824	2	0.064
Baseline P-T181-tau	3.41±0.97	3.63±0.80	3.30±0.85	1.307	2	0.275
Week-28 P-T181-tau	3.26±0.82*	3.73±0.66	3.23±0.78*	4.900	2	**0.009**
Baseline P-S396-tau	2.83±0.87	3.03±1.02	2.92±0.86	0.444	2	0.643
Week-28 P-S396-tau	2.75±0.85	3.13±0.98	2.65±0.86	2.770	2	0.067
Baseline NRGN	5.09±1.05	5.20±0.90	4.92±0.75	0.811	2	0.447
Week-28 NRGN	4.79±1.26	5.14±1.13	4.67±1.35	1.340	2	0.266
Baseline REST	5.74±0.91	5.78±1.06	5.45±0.68	1.476	2	0.233
Week-28 REST	5.71±0.88	5.73±0.78	5.40±0.91	1.597	2	0.207

Aβ_42_, amyloid-β 1-42; ADAS-cog+, AD assessment scale-cognitive subscale modified; MMSE, Mini-Mental State Examination; nL, natural log; NRGN, Neurogranin; P-T181-tau, tau phosphorylated at threonine 181; P-S396-tau, tau phosphorylated at serine 396; REST, repressor element 1-silencing transcription factor. Chi-Square (χ^2^) and ANOVA (F) analyses were employed. *p < 0.05 versus donepezil group (ANOVA).

**Table 3:** Effects of Cerebrolysin®, donepezil, and combination therapy on plasma neuronal-derived extracellular vesicles (NDEV) biomarkers in AD: Changes from baseline to week 28 (study endpoint)

		Baseline		Week 28	
	*N*	Baseline Score (pg/mL)	LS mean change±SE (nL data)	Treatment Difference (95% CI)	*p*
**Aß_**42**_**
Cerebrolysin®	37	17.10	–0.079±0.172	0.558 (0.061/1.055)	**0.028**
Donepezil	36	22.38	–0.097±0.176	0.540 (0.035/1.045)	**0.036**
Combination	35	14.90	–0.638±0.180
**Total-tau**
Cerebrolysin®	37	85.78	–0.141±0.129	-0.445 (–0.811/–0.079)	**0.018**
Donepezil	35	96.37	0.304±0.132
Combination	35	108.46	–0.052±0.132	–0.356 (–0.727/0.015)	0.060
**P-T181-tau**
Cerebrolysin®	38	45.99	–0.170±0.108	–0.374 (–0.683/–0.065)	**0.018**
Donepezil	36	53.79	0.204±0.112
Combination	36	39.23	–0.158±0.112	–0.362 (–0.677/–0.046)	**0.025**
**P-S396-tau**
Cerebrolysin®	36	25.51	–0.110±0.132	–0.261 (–0.638/0.116)	0.172
Donepezil	34	36.49	0.152±0.132
Combination	35	25.90	–0.284±0.134	–0.435 (–0.814/–0.057)	**0.025**
**NRGN**
Cerebrolysin®	38	266.47	–0.292±0.167	–0.262 (–0.737/0.213)	0.277
Donepezil	36	304.39	–0.031±0.172
Combination	36	224.38	–0.281±0.172	–0.250 (–0.735/0.234)	0.308
**REST**
Cerebrolysin®	37	444.88	–0.049±0.098	–0.027 (–0.307/0.253)	0.850
Donepezil	34	480.82	–0.022±0.102
Combination	35	316.01	–0.104±0.100	–0.082 (–0.368/0.203)	0.570

Aβ_42_, amyloid-β 1-42; nL, natural log; NRGN, Neurogranin; P-T181-tau, tau phosphorylated at threonine 181; P-S396-tau, tau phosphorylated at serine 396; REST, repressor element 1-silencing transcription factor. ANCOVA analysis was used.

Sample size estimates have been made taking into account the primary endpoints, i.e., baseline levels of NDEV biomarkers of primary interest (Aβ_42_ and P-T181-tau) for comparisons between AD patients and controls, and changes from baseline in these parameters for differences in treatment effects. According to results of a previous study [10], it was calculated that sample sizes of 20 controls and 100 patients would allow to show significant group differences with 80% power for four-fold and two-fold differences in NDEV levels of Aβ_42_ and P-T181-tau, respectively. With a sample size of 33 AD cases per treatment group, the power to detect treatment differences in CD81-adjusted Aβ_42_ and P-T181-tau changes from baseline of 4 pg/ml and 12 pg/ml (approximately 25% of average baseline values), respectively, was around 80%.

## RESULTS

As shown in Table 1, average age and the distribution by gender were similar in control and AD samples; while significant group differences (*p* < 0.001) were observed for MMSE and ADAS-cog+scores of cognitive performance. Average normalized NDEV CD81 values were similar in AD and control cases (0.39 pg/ml versus 0.32 pg/ml) and showed no differences by *APOE4* status and AD severity. Compared to controls, AD patients had significantly higher NDEV levels of Aβ_42_ (*p* < 0.05; average CD81-adjusted levels: 19.60 pg/ml versus 5.00 pg/ml), total-tau (*p* < 0.01; CD81-adjusted levels: 110.58 pg/ml versus 38.71 pg/ml), P-T181-tau (*p* < 0.001; CD81-adjusted levels: 48.79 pg/ml versus 15.10 pg/ml), and P-S396-tau (*p* < 0.05; CD81-adjusted levels: 32.63 pg/ml versus 17.78 pg/ml), and significantly lower NDEV concentrations of NRGN (*p* < 0.01; CD81-adjusted levels: 288.02 pg/ml versus 498.61 pg/ml) (Table 1). No significant differences were found between AD and control cases for NDEV REST (*p* = 0.078; CD81-adjusted levels: 418.10 pg/ml versus 529.92 pg/ml) (Table 1). The levels of all these NDE markers did not show any significant correlation with age, nor significant differences between cases with and without *APOE4*.

Significant severity-related changes were found for plasma NDEV levels of Aβ_42_ (*p* < 0.05), total tau (*p* < 0.001), P-T181-tau (*p* < 0.001), P-S936-tau (*p* < 0.05), NRGN (*p* < 0.001), and REST (*p* < 0.01). NDE Aβ_42_ values were consistently elevated in mild-to-moderate (*p* < 0.05; CD81-adjusted levels: 18.05 pg/ml) and in advanced AD stages (*p* < 0.05; CD81-adjusted levels: 23.06 pg/ml) but showed no differences between AD subgroups (Fig. 1a). NDEV total tau levels increased with disease severity (Fig. 1b), were significantly elevated in mild-to-moderate AD (*p* < 0.01; CD81-adjusted levels: 97.16 pg/ml) and in advanced AD (*p* < 0.001; CD81-adjusted levels: 140.85 pg/ml) compared with controls and were higher in advanced AD cases than in mild-to-moderate AD patients (*p* < 0.05). Concentrations of NDEV P-T181-tau were significantly increased (*p* < 0.001) with respect to controls in mild-to-moderate (CD81-adjusted levels: 44.28 pg/ml) and advanced AD (CD81-adjusted levels: 59.21 pg/ml), and did not show significant severity-related differences in AD cases (Fig. 1c). Average NDEV P-S396-tau levels were significantly higher (*p* < 0.05) in mild-to-moderate AD (CD81-adjusted levels: 34.13 pg/ml), but not in advanced AD (CD81-adjusted levels: 28.82 pg/ml), than in controls (Fig. 1d). NDEV levels of NRGN and REST exhibited a similar pattern of decreased levels in mild-to-moderate AD patients (NRGN CD81-adjusted levels: 233.23 pg/ml; REST CD81-adjusted levels: 367.93 pg/ml) compared with controls (*p* < 0.001 and *p* < 0.05, respectively) and advanced AD (*p* < 0.01 both) (Fig. 1e,f).

In the population of AD patients, and after controlling for the influence of covariates, NDEV levels of total tau, NRGN, and REST correlated negatively with MMSE (Total tau: *r = –*0.267, *p* < 0.01; NRGN: *r = –*0.262, *p* < 0.01; REST: *r = –*0.194, *p* < 0.05) and ADCS-ADL scores (Total tau: *r = –*0.226, *p* < 0.05; NRGN: *r = –*0.257, *p* < 0.01; REST: *r = –*0.269, *p* < 0.01), and showed positive correlations with ADAS-cog+scores (Total tau: *r* = 0.237, *p* < 0.05; NRGN: *r* = 0.293, *p* < 0.01; REST: *r* = 0.225, *p* < 0.05). These correlations indicate that increases in NDEV total-tau, NRGN, and REST are associated with greater impairments in cognition and functioning. However, since such correlations were not significant in mild-to-moderate AD, they could simple be a reflection of the progressive elevations of these blood markers with increasing AD severity. In fact, when controlling for disease severity as well, the only correlation that remained significant was a weak negative correlation between total tau values and MMSE scores (*r = –*0.189, *p* < 0.05). In addition, the levels of NDEV total tau and plasma TNF-α were positively correlated in AD cases (*r* = 0.254, *p* < 0.01), and NDE Aβ_42_ and P-T181-tau showed significant negative correlations with serum BDNF (*r = –*274 and *r = –*250, respectively, *p* < 0.05) in patients with mild-to-moderate AD.

Clinical characteristics and baseline levels of CD81-adjusted NDEV biomarkers were similar in the three treatment subgroups of AD patients and are summarized in Table 2. The combination therapy induced a significant reduction (*p* < 0.05) of NDEV Aβ_42_ from baseline to week-28 in comparison with Cerebrolysin® and donepezil monotherapies (Table 3, Fig. 2a). Estimated reductions from baseline in Aβ_42_ levels were around 10% after Cerebrolysin® and donepezil treatment, and more than 40% in patients on combination therapy. In patients treated with Cerebrolysin® alone and/or combined with donepezil, NDEV total-tau, P-T181-tau, and P-S396-tau concentrations decreased significantly from baseline to week-28 (*p* < 0.05) as compared to patients on donepezil monotherapy (Table 3, Fig. 2b–d). Changes from baseline in total-tau levels represent a 20% reduction in the Cerebrolysin® group and a 30% increase in the donepezil group. Regarding changes from baseline in P-T181-tau values, Cerebrolysin® and combination therapy induced an approximately 25% decrease and donepezil-treated patients showed a 15% increase. Combination therapy reduced NDEV P-S396-tau by more than 30%, while P-S396-tau levels increased by 15% compared to baseline in patients on donepezil monotherapy. Changes from baseline in NDEV total-tau and ADAS-cog+correlated positively (*r* = 0.231, *p* = 0.019), this correlation indicating that treatment-induced reductions in total tau are associated with cognitive improvements. No significant treatment differences were found for changes from baseline in NDEV NRGN and REST concentrations (Table 3). Decreases in the levels of these synaptic markers after treatment were significant for NRGN in the whole trial population (pairwise comparison: *F* = 4.206, *p* = 0.043), and were more pronounced in Cerebrolysin-treated cases (pairwise comparison: *F* = 5.060, *p* = 0.028).

## DISCUSSION

Two recent meta-analyses support determinations of the levels of Aβ_42_, total-tau, P-T181-tau, P-S396-tau, and NRGN in plasma NDEVs as effective diagnostic and cognitive blood biomarkers for AD [7, 16]. A previous investigation demonstrated that elevated Aβ_42_, P-T181-tau, and P-S936-tau, and reduced NRGN and REST levels in plasma NDEV distinguished control subjects from mild to moderate AD patients and from patients with mild cognitive impairment (MCI) who progressed to AD with a high sensitivity [10]. Enhanced NDEV levels of P-T181-tau, P-S396-tau, and Aβ_42_ were also found to predict the development of AD up to 10 years before clinical diagnosis [11] while increased NDEV Aβ_42_ levels in MCI subjects have been associated with an 8.5-fold greater risk of developing AD dementia [20]. Other authors reported that plasma NDEV Aβ_42_, total tau, P-T181-tau, and NRGN had a similar diagnostic power as their CSF markers in differentiating AD from controls [12, 26]. Moreover, reduced NDEV levels of NRGN [10, 17, 18, 26] and REST [10, 17, 29] with respect to controls were observed at both preclinical and dementia AD stages as well. All these studies, however, included mainly patients with mild AD, and do not allow the assessment of changes in the levels of these blood biomarkers or in their associations with cognition through the clinical course of AD dementia. Therefore, the first goal of the present investigation was to determine plasma NDEV levels of these six markers and their correlations with cognition and functioning in patients with mild-to-moderate and advanced AD and in age-matched controls.

Our results show significant differences between controls and AD for all blood markers except REST (Table 1). In line with data reported by most studies [10–12, 16–20, 26], we found that plasma NDEV levels of Aβ_42_, total-tau, P-T181-tau, and P-S393-tau are higher and those of NRGN and REST lower in patients with mild-to-moderate AD than in control subjects (Fig. 1a–f). In the AD patient population, all NDEV markers except P-S396-tau followed a common pattern of progressive increase with dementia severity that was significant for total tau, NRGN, and REST, but not for Aß_42_ and P-T181-tau (Fig. 1a–c, e, f). In contrast, NDEV levels of P-S396-tau appear to decrease with dementia severity, although not significantly (Fig. 1d). The lack of previous studies does not allow us to contrast our findings in advanced AD, and in particular the lack of differences with respect to the control cases observed for the levels of P-S396-tau, NRGN, and REST (Fig. 1d–f).

The absence of significant changes related to AD severity that we observed for Aβ_42_ and P-T181-tau seem to indicate that major changes in these blood markers occur early in AD pathology, as supported by the findings that alterations in NDEV and CSF levels of both parameters are similar in the preclinical and dementia stages of AD [10, 11, 18, 58]. NDE levels of Aß_42_ and P-T181-tau correlated negatively with serum BDNF values in mild-to-moderate AD cases, but did not show any significant correlation with measures of cognition and functioning in AD. The negative associations of BDNF with Aβ_42_ and P-T181-tau suggest that neurotrophic factors could counteract AD pathogenic factors. Reduced serum BDNF in preclinical AD [58] and in AD dementia *APOE4* + females [59], and significant positive correlations of serum BDNF with CSF Aβ_42_ [58] and with MMSE [59] in AD have been reported. Other authors also found a lack of correlation between NDE Aβ_42_ and P-T181-tau levels and MMSE and ADAS-cog indices of cognition [11, 18].

Elevations in NDEV total tau, NRGN and REST levels with advancing AD severity and their associations with cognitive and functional decline might reflect the aggravation of neuronal and synaptic damage during the course of AD dementia. In any case, the enhanced expression of total tau, NRGN and REST in plasma NDEVs from advanced AD cases is not due to severity-related variations in CD81 + NDEV counts, and needs to be replicated and further investigated. Increases in plasma NDEV levels of total tau and reductions in NRGN and REST along the AD continuum (C-MCI-AD) have been shown in several studies [10, 12, 21, 22, 26]. In our AD population, total tau was the only plasma NDEV biomarker associated to cognitive impairment with independence of the dementia severity. Shi et al. [22] reported a significant negative correlation of plasma, but not NDEV, tau with MMSE scores in mild-to-moderate AD. Nam et al. [21] also found that higher serum and NDEV levels of total tau predicted a faster deterioration of cognition and global functioning in mild AD. The correlation of NDEV total tau with plasma TNF-α suggests an interaction of these neurodegenerative and inflammatory factors in AD pathology. Circulating levels of TNF-α were found to be enhanced and negatively associated with the expression of the trophic factor IGF-1 in AD and MCI patients [60], and a recent investigation demonstrated that plasma and CSF analytes of the TNF signaling pathway correlate with CSF total tau in MCI patients [61].

Characterization of the behavior of these plasma NDEV biomarkers throughout the entire AD continuum could be relevant for the design and monitoring of therapeutic interventions in clinical trials with MCI and AD patients. Results of a recent 20-week clinical trial showed that plasma NDEV levels of Aβ_42_, NRGN, and other synaptic markers were not modulated by the treatment with growth hormone releasing hormone in MCI patients [52]. Another study found that a mindfulness-based stress reduction intervention increased plasma REST levels compared with a placebo intervention in older adults with psychiatric risk factors for AD, and that elevated REST levels were associated with a reduction in psychiatric symptoms related to stress and AD risk [26]. The protocol of a clinical trial aimed at investigating the associations of plasma NDEV NRGN with changes in brain structures and cognition in early AD stages has also been published recently [62].

In the present investigation, we found for the first time that Cerebrolysin® and donepezil induced a synergistic reduction in plasma NDE Aβ_42_, and that Cerebrolysin® and/or combination therapy reduced NDEV levels of total tau, P-T181-tau, and P-S396-tau with respect to donepezil monotherapy in mild to moderately severe AD patients. Changes in plasma NDEV expression of these biomarkers could reflect neuroprotective and anti-degenerative activities of Cerebrolysin® and combination therapy resulting in diminutions in Aβ_42_ and tau production and tau phosphorylation in neurons, and eventually in less neuronal loss. The finding that treatment-related decreases in NDEV total tau levels were associated with improvements in cognitive performance as assessed by ADAS-cog+highlights the relevance of this biomarker for cognition in AD and supports the possibility that Cerebrolysin® and combination therapy effects on tau expression and cognition are mediated by direct actions on the brain. Correlations at baseline of this biomarker with MMSE (*r = –*0.278, *p* = 0.004) and ADAS-cog+(*r* = 0.266, *p* = 0.006) in the RCT and with MMSE in the NDEV Study are also indicating a negative relationship between NDEV total tau and cognition in our AD population. In agreement with these findings, other authors observed a faster deterioration in mild AD patients with elevated circulating levels of total tau [21]. Therefore, it seems that decreases in NDEV total tau are contributing to the amelioration of cognitive deficits in AD patients.

The mechanisms by which Cerebrolysin® and donepezil may influence Aß_42_ and tau expression, and tau phosphorylation had not been investigated in AD patients but increases in serum BDNF and reductions in TNF-α induced by Cerebrolysin® and the combination therapy [2, 50, 63, 64] might contribute to the decreases in NDE biomarkers observed in this study. We previously found that Cerebrolysin® increased serum BNDF levels and that Cerebrolysin® and donepezil acted synergistically to augment and prolong the increase of BDNF in patients of the RCT [64]. Here we observed negative associations between serum BDNF and NDEV Aβ_42_ and P-T181-tau in AD samples of both the NDEV Study and the RCT (Aβ_42_: *r = –*0.207, *p* = 0.034; P-T181-tau: *r = –*0.218, *p* = 0.024), suggesting that an interaction between increases in serum BDNF and reductions in NDEV Aβ_42_ and P-T181-tau might exist and has to be further investigated. The recent finding of a positive correlation between serum BDNF and CSF Aβ_42_ is supporting our observations and indicates that elevated serum BDNF could represent a protective factor against Aβ pathology [58]. Thus, increases in serum BDNF might have some influence on the reductions in NDEV Aβ_42_ and P-T181-tau produced by Cerebrolysin® and the combination therapy. On the other hand, Cerebrolysin® and the combination therapy were also found to reduce circulating TNF-α in AD patients [50, 63], and baseline NDEV total tau showed positive correlations with plasma TNF-α in the NDEV Study and in the RCT (*r* = 0.299, *p* = 0.002). Therefore, reductions in plasma TNF-α could influence to some extent the decrease of total tau in plasma NDEVs of patients treated with Cerebrolysin®, and this potential interaction deserves future investigation. Finally, our results are consistent with and supported by experimental studies demonstrating that Cerebrolysin® reduces Aβ and tau pathology [35–42] and that donepezil decreases Aβ and increases tau expression [43–47].

Based on the results of the present investigation, we conclude the following:

1) In agreement with previous studies, we showed that plasma NDEV levels of Aβ_42_, total tau, P-T181-tau, and P-S393-tau are higher and those of NRGN and REST are lower in mild-to-moderate AD patients than in controls;
2) This report is the first to show that:
  a. NDEV total tau, NRGN, and REST increase with AD severity and in association with cognitive and functional decline;
  b. NDEV Aβ_42_ and P-T181-tau correlate negatively with serum BDNF;
  c. Higher levels of NDE total-tau are associated with elevated plasma TNF-α and with worse cognition in AD patients;
3) We also demonstrated here for the first time that:
  a. Cerebrolysin® and donepezil reduce NDEV Aβ_42_ synergistically;
  b. Compared with donepezil monotherapy, Cerebrolysin® treatment induced decreases in the plasma NDEV levels of total tau, P-T181-tau, and P-S396-tau.
4) This is one of the first studies to support the use of neuronal-derived extracellular vesicles as effective tools to monitor AD the clinical continuum and AD drug therapy interventions. Future studies are warranted to confirm our findings on changes in plasma NDEV biomarkers in advanced AD and after treatment with Cerebrolysin® and donepezil, as well as on correlations of these biomarkers with the neurotrophic and inflammatory factors BDNF and TNF-α, because this is the first report on these topics. Future research should also overcome the main limitations of our investigation, such as the lack of inclusion of MCI subjects in the NDEV Study and of a placebo group in the RCT, and the absence of CSF and plasma AD biomarkers determinations in cases and controls.

## References

[1] Cummings J (2019) The role of biomarkers in Alzheimer’s disease drug development. Adv Exp Med Biol 1118, 29–61. 30747416 10.1007/978-3-030-05542-4_2 PMC6750734

[2] Gavrilova SI , Alvarez A (2021) Cerebrolysin in the therapy of mild cognitive impairment and dementia due to Alzheimer’s disease: 30 years of clinical use. Med Res Rev 41, 2775–2803. 32808294 10.1002/med.21722

[3] Jack CR Jr , Bennett DA , Blennow K , Carrillo MC , Dunn B , Haeberlein SB , Holtzman DM , Jagust W , Jessen F , Karlawish J , Liu E , Molinuevo JL , Montine T , Phelps C , Rankin KP , Rowe CC , Scheltens P , Siemers E , Snyder HM , Sperling R ; Contributors (2018) NIA-AA Research Framework: Toward a biological definition of Alzheimer’s disease. Alzheimers Dement 14, 535–562. 29653606 10.1016/j.jalz.2018.02.018 PMC5958625

[4] Nelson PT , Braak H , Markesbery WR (2009) Neuropathology and cognitive impairment in Alzheimer disease: A complex but coherent relationship. J Neuropathol Exp Neurol 68, 1–14. 19104448 10.1097/NEN.0b013e3181919a48 PMC2692822

[5] Serrano-Pozo A , Frosch MP , Masliah E , Hyman BT (2011) Neuropathological alterations in Alzheimer disease. Cold Spring Harb Perspect Med 1, a006189. 22229116 10.1101/cshperspect.a006189 PMC3234452

[6] Hampel H , Goetzl EJ , Kapogiannis D , Lista S , Vergallo A (2019) Biomarker-drug and liquid biopsy co-development for disease staging and targeted therapy: Cornerstones for Alzheimer’s precision medicine and pharmacology. Front Pharmacol 10, 310. 30984002 10.3389/fphar.2019.00310 PMC6450260

[7] Kim KY , Shin KY , Chang KA (2021) Brain-derived exosomal proteins as effective biomarkers for Alzheimer’s disease: A systematic review and meta-analysis. Biomolecules 11, 980. 34356604 10.3390/biom11070980 PMC8301985

[8] Song Z , Xu Y , Deng W , Zhang L , Zhu H , Yu P , Qu Y , Zhao W , Han Y , Qin C (2020) Brain derived exosomes are a double-edged sword in Alzheimer’s disease. Front Mol Neurosci 13, 79. 32547364 10.3389/fnmol.2020.00079 PMC7274346

[9] Watson LS , Hamlett ED , Stone TD , Sims-Robinson C (2019) Neuronally derived extracellular vesicles: An emerging tool for understanding Alzheimer’s disease. Mol Neurodegener 14, 22. 31182115 10.1186/s13024-019-0317-5 PMC6558712

[10] Winston CN , Goetzl EJ , Akers JC , Carter BS , Rockenstein EM , Galasko D , Masliah E , Rissman RA (2016) Prediction of conversion from mild cognitive impairment to dementia with neuronally derived blood exosome protein profile. Alzheimers Dement (Amst) 3, 63–72. 27408937 10.1016/j.dadm.2016.04.001 PMC4925777

[11] Fiandaca MS , Kapogiannis D , Mapstone M , Boxer A , Eitan E , Schwartz JB , Abner EL , Petersen RC , Federoff HJ , Miller BL , Goetzl EJ (2015) Identification of preclinical Alzheimer’s disease by a profile of pathogenic proteins in neurally derived blood exosomes: A case-control study. Alzheimers Dement 11, 600–607.e1. 25130657 10.1016/j.jalz.2014.06.008 PMC4329112

[12] Jia L , Qiu Q , Zhang H , Chu L , Du Y , Zhang J , Zhou C , Liang F , Shi S , Wang S , Qin W , Wang Q , Li F , Wang Q , Li Y , Shen L , Wei Y , Jia J (2019) Concordance between the assessment of Aβ42, T-tau, and P-T181-tau in peripheral blood neuronal-derived exosomes and cerebrospinal fluid. Alzheimers Dement 15, 1071–1080. 31422798 10.1016/j.jalz.2019.05.002

[13] Miyoshi E , Bilousova T , Melnik M , Fakhrutdinov D , Poon WW , Vinters HV , Miller CA , Corrada M , Kawas C , Bohannan R , Caraway C , Elias C , Maina KN , Campagna JJ , John V , Gylys KH (2021) Exosomal tau with seeding activity is released from Alzheimer’s disease synapses, and seeding potential is associated with amyloid beta. Lab Invest 101, 1605–1617. 34462532 10.1038/s41374-021-00644-z PMC8590975

[14] Sardar Sinha M , Ansell-Schultz A , Civitelli L , Hildesjö C , Larsson M , Lannfelt L , Ingelsson M , Hallbeck M (2018) Alzheimer’s disease pathology propagation by exosomes containing toxic amyloid-beta oligomers. Acta Neuropathol 136, 41–56. 29934873 10.1007/s00401-018-1868-1 PMC6015111

[15] Badhwar A , Haqqani AS (2020) Biomarker potential of brain-secreted extracellular vesicles in blood in Alzheimer’s disease. Alzheimers Dement (Amst) 12, e12001. 32211497 10.1002/dad2.12001 PMC7085285

[16] Liu W , Lin H , He X , Chen L , Dai Y , Jia W , Xue X , Tao J , Chen L (2020) Neurogranin as a cognitive biomarker in cerebrospinal fluid and blood exosomes for Alzheimer’s disease and mild cognitive impairment. Transl Psychiatry 10, 125. 32350238 10.1038/s41398-020-0801-2 PMC7190828

[17] Abner EL , Jicha GA , Shaw LM , Trojanowski JQ , Goetzl EJ (2016) Plasma neuronal exosomal levels of Alzheimer’s disease biomarkers in normal aging. Ann Clin Transl Neurol 3, 399–403. 27231710 10.1002/acn3.309 PMC4863753

[18] Goetzl EJ , Kapogiannis D , Schwartz JB , Lobach IV , Goetzl L , Abner EL , Jicha GA , Karydas AM , Boxer A , Miller BL (2016) Decreased synaptic proteins in neuronal exosomes of frontotemporal dementia and Alzheimer’s disease. FASEB J 30, 4141–4148. 27601437 10.1096/fj.201600816R PMC5102122

[19] Lim CZJ , Zhang Y , Chen Y , Zhao H , Stephenson MC , Ho NRY , Chen Y , Chung J , Reilhac A , Loh TP , Chen CLH , Shao H (2019) Subtyping of circulating exosome-bound amyloid β reflects brain plaque deposition. Nat Commun 10, 1144. 30850633 10.1038/s41467-019-09030-2 PMC6408581

[20] Zhao A , Li Y , Yan Y , Qiu Y , Li B , Xu W , Wang Y , Liu J , Deng Y (2020) Increased prediction value of biomarker combinations for the conversion of mild cognitive impairment to Alzheimer’s dementia. Transl Neurodegener 9, 30. 32741361 10.1186/s40035-020-00210-5 PMC7397685

[21] Nam E , Lee YB , Moon C , Chang KA (2020) Serum tau proteins as potential biomarkers for the assessment of Alzheimer’s disease progression. Int J Mol Sci 21, 5007. 32679907 10.3390/ijms21145007 PMC7404390

[22] Shi M , Kovac A , Korff A , Cook TJ , Ginghina C , Bullock KM , Yang L , Stewart T , Zheng D , Aro P , Atik A , Kerr KF , Zabetian CP , Peskind ER , Hu SC , Quinn JF , Galasko DR , Montine TJ , Banks WA , Zhang J (2016) CNS tau efflux via exosomes is likely increased in Parkinson’s disease but not in Alzheimer’s disease. Alzheimers Dement 12, 1125–1131. 27234211 10.1016/j.jalz.2016.04.003 PMC5107127

[23] Guix FX , Corbett GT , Cha DJ , Mustapic M , Liu W , Mengel D , Chen Z , Aikawa E , Young-Pearse T , Kapogiannis D , Selkoe DJ , Walsh DM (2018) Detection of aggregation-competent tau in neuron-derived extracellular vesicles. Int J Mol Sci 19, 663. 29495441 10.3390/ijms19030663 PMC5877524

[24] Kvartsberg H , Lashley T , Murray CE , Brinkmalm G , Cullen NC , Höglund K , Zetterberg H , Blennow K , Portelius E (2019) The intact postsynaptic protein neurogranin is reduced in brain tissue from patients with familial and sporadic Alzheimer’s disease. Acta Neuropathol 137, 89–102. 30244311 10.1007/s00401-018-1910-3 PMC6338696

[25] Willemse EAJ , Sieben A , Somers C , Vermeiren Y , De Roeck N , Timmers M , Van Broeckhoven C , De Vil B , Cras P , De Deyn PP , Martin JJ , Teunissen CE , Engelborghs S , Bjerke M (2021) Neurogranin as biomarker in CSF is non-specific to Alzheimer’s disease dementia. Neurobiol Aging 108, 99–109. 34551375 10.1016/j.neurobiolaging.2021.08.002

[26] Jia L , Zhu M , Kong C , Pang Y , Zhang H , Qiu Q , Wei C , Tang Y , Wang Q , Li Y , Li T , Li F , Wang Q , Li Y , Wei Y , Jia J (2021) Blood neuro-exosomal synaptic proteins predict Alzheimer’s disease at the asymptomatic stage. Alzheimers Dement 17, 49–60. 32776690 10.1002/alz.12166 PMC7984076

[27] Lu T , Aron L , Zullo J , Pan Y , Kim H , Chen Y , Yang TH , Kim HM , Drake D , Liu XS , Bennett DA , Colaiacovo MP , Yankner BA (2014) REST and stress resistance in ageing and Alzheimer’s disease. Nature 507, 448–454. 24670762 10.1038/nature13163 PMC4110979

[28] Ashton NJ , Hye A , Leckey CA , Jones AR , Gardner A , Elliott C , Wetherell JL , Lenze EJ , Killick R , Marchant NL (2017) Plasma REST: A novel candidate biomarker of Alzheimer’s disease is modified by psychological intervention in an at-risk population. Transl Psychiatry 7, e1148. 28585932 10.1038/tp.2017.113 PMC5537638

[29] Goetzl EJ , Boxer A , Schwartz JB , Abner EL , Petersen RC , Miller BL , Carlson OD , Mustapic M , Kapogiannis D (2015) Low neural exosomal levels of cellular survival factors in Alzheimer’s disease. Ann Clin Transl Neurol 2, 769–773. 26273689 10.1002/acn3.211 PMC4531059

[30] Ramírez-Cuapio FL , Torres-Ramos MA , Orozco-Ibarra M , Acosta I , Sosa-Ortiz AL (2020) Serum repressor element-1 silencing transcription factor levels in Alzheimer’s patients from a National Institute of Health in Mexico City, elderly and young controls. Rev Invest Clin 73, 017–022. 33053565 10.24875/RIC.20000089

[31] Ngolab J , Trinh I , Rockenstein E , Mante M , Florio J , Trejo M , Masliah D , Adame A , Masliah E , Rissman RA (2017) Brain-derived exosomes from dementia with Lewy bodies propagate α-synuclein pathology. Acta Neuropathol Commun 5, 46. 28599681 10.1186/s40478-017-0445-5 PMC5466770

[32] Winston CN , Goetzl EJ , Baker LD , Vitiello MV , Rissman RA (2018) Growth hormone-releasing hormone modulation of neuronal exosome biomarkers in mild cognitive impairment. J Alzheimers Dis 66, 971–981. 30372675 10.3233/JAD-180302 PMC6487872

[33] Alvarez XA , Cacabelos R , Sampedro C , Couceiro V , Aleixandre M , Vargas M , Linares C , Granizo E , García-Fantini M , Baurecht W , Doppler E , Moessler H (2011) Combination treatment in Alzheimer’s disease: Results of a randomized, controlled trial with cerebrolysin and donepezil. Curr Alzheimer Res 8, 583–591. 21679156 10.2174/156720511796391863

[34] Gauthier S , Proaño JV , Jia J , Froelich L , Vester JC , Doppler E (2015) Cerebrolysin in mild-to-moderate Alzheimer’s disease: Ameta-analysis of randomized controlled clinical trials. DementGeriatr Cogn Disord 39, 332–347. 10.1159/000377672 25832905

[35] Rockenstein E , Torrance M , Mante M , Adame A , Paulino A , Rose JB , Crews L , Moessler H , Masliah E (2006) Cerebrolysin decreasesamyloid-beta production by regulating amyloid protein precursormaturation in a transgenic model of Alzheimer’s disease. JNeurosci Res 83, 1252–1261. 16511867 10.1002/jnr.20818

[36] Rockenstein E , Ubhi K , Pham E , Michael S , Doppler E , Novak P , Inglis C , Mante M , Adame A , Alvarez XA , Moessler H , Masliah E (2011) Beneficial effects of a neurotrophic peptidergic mixture persist for a prolonged period following treatment interruption in a transgenic model of Alzheimer’s disease. J Neurosci Res 89, 1812–1821. 21793038 10.1002/jnr.22712 PMC3171597

[37] Sharma HS , Muresanu DF , Lafuente JV , Patnaik R , Tian ZR , Ozkizilcik A , Castellani RJ , Mössler H , Sharma A (2018) Co-administration of TiO2 nanowired mesenchymal stem cells with cerebrolysin potentiates neprilysin level and reduces brain pathology in Alzheimer’s disease. Mol Neurobiol 55, 300–311. 28844104 10.1007/s12035-017-0742-9

[38] Zhang Y , Chopp M , Zhang ZG , Zhang Y , Zhang L , Lu M , Zhang T , Winter S , Doppler E , Brandstäetter H , Mahmood A , Xiong Y (2019) Cerebrolysin reduces astrogliosis and axonal injury and enhances neurogenesis in rats after closed head injury. Neurorehabil Neural Repair 33, 15–26. 30499355 10.1177/1545968318809916

[39] Xing S , Zhang J , Dang C , Liu G , Zhang Y , Li J , Fan Y , Pei Z , Zeng J (2014) Cerebrolysin reduces amyloid-β deposits, apoptosis and autophagy in the thalamus and improves functional recovery after cortical infarction. J Neurol Sci 337, 104–111. 24315581 10.1016/j.jns.2013.11.028

[40] Ubhi K , Rockenstein E , Doppler E , Mante M , Adame A , Patrick C , Trejo M , Crews L , Paulino A , Moessler H , Masliah E (2009) Neurofibrillary and neurodegenerative pathology in APP-transgenic mice injected with AAV2-mutant TAU: Neuroprotective effects of Cerebrolysin. Acta Neuropathol 117, 699–712. 19252918 10.1007/s00401-009-0505-4 PMC3049872

[41] Rockenstein E , Ubhi K , Trejo M , Mante M , Patrick C , Adame A , Novak P , Jech M , Doppler E , Moessler H , Masliah E (2014) Cerebrolysin™ efficacy in a transgenic model of tauopathy: Role in regulation of mitochondrial structure. BMC Neurosci 15, 90. 25047000 10.1186/1471-2202-15-90 PMC4122761

[42] Rockenstein E , Ubhi K , Mante M , Florio J , Adame A , Winter S , Brandstaetter H , Meier D , Masliah E (2015) Neuroprotective effects of Cerebrolysin in triple repeat Tau transgenic model of Pick’s disease and fronto-temporal tauopathies. BMC Neurosci 16, 85. 26611895 10.1186/s12868-015-0218-7 PMC4662012

[43] Ye CY , Lei Y , Tang XC , Zhang HY (2015) Donepezil attenuates Aβ-associated mitochondrial dysfunction and reduces mitochondrial Aβ accumulation *in vivo* and *in vitro*. Neuropharmacology 95, 29–36. 25744714 10.1016/j.neuropharm.2015.02.020

[44] Takada-Takatori Y , Nakagawa S , Kimata R , Nao Y , Mizukawa Y , Urushidani T , Izumi Y , Akaike A , Tsuchida K , Kume T (2019) Donepezil modulates amyloid precursor protein endocytosis and reduction by up-regulation of SNX33 expression in primary cortical neurons. Sci Rep 9, 11922. 31417133 10.1038/s41598-019-47462-4 PMC6695423

[45] Easton A , Sankaranarayanan S , Tanghe A , Terwel D , Lin AX , Hoque N , Bourin C , Gu H , Ahlijanian M , Bristow L (2013) Effects of sub-chronic donepezil on brain Abeta and cognition in a mouse model of Alzheimer’s disease. Psychopharmacology (Berl) 230, 279–289. 23783773 10.1007/s00213-013-3152-3

[46] Ma Y , Ji J , Li G , Yang S , Pan S (2018) Effects of donepezil on cognitive functions and the expression level of β-amyloid in peripheral blood of patients with Alzheimer’s disease. Exp Ther Med 15, 1875–1878. 29434777 10.3892/etm.2017.5613 PMC5776632

[47] Hellström-Lindahl E , Moore H , Nordberg A (2000) Increased levels of tau protein in SH-SY5Y cells after treatment with cholinesterase inhibitors and nicotinic agonists. J Neurochem 74, 777–784. 10646530 10.1046/j.1471-4159.2000.740777.x

[48] McKhann G , Drachman D , Folstein M , Katzman R , Price D , Stadlan EM (1984) Clinical diagnosis of Alzheimer’s disease: Report of NINCDS-ADRDA Work Group. Neurology 34, 939–944. 6610841 10.1212/wnl.34.7.939

[49] American Psychiatric Association (2013) Diagnostic and Statistical Manual of Mental Disorders (5th Ed). American Psychiatric Publishing, Arlington, VA.

[50] Alvarez A , Alvarez I , Martinez A , Romero I , Benito C , Suarez I , Mourente S , Fantini M , Figueroa J , Aleixandre M , Linares C , Muresanu D , Winter S , Moessler H (2020) Serum VEGF predicts clinical improvement induced by Cerebrolysin plus donepezil in patients with advanced Alzheimer’s disease. Int J Neuropsychopharmacol 23, 581–586. 32640027 10.1093/ijnp/pyaa046 PMC7710915

[51] Hamilton M (1967) Development of a rating scale for primary depressive illness. Br J Soc Clin Psychol 6, 278–296. 6080235 10.1111/j.2044-8260.1967.tb00530.x

[52] Winston CN , Romero HK , Ellisman M , Nauss S , Julovich DA , Conger T , Hall JR , Campana W , O’Bryant SE , Nievergelt CM , Baker DG , Risbrough VB , Rissman RA (2019) Assessing neuronal and astrocyte derived exosomes from individuals with mild traumatic brain injury for markers of neurodegeneration and cytotoxic activity. Front Neurosci 13, 1005. 31680797 10.3389/fnins.2019.01005 PMC6797846

[53] Mustapic M , Eitan E , Werner JK Jr. , Berkowitz ST , Lazaropoulos MP , Tran J , Goetzl EJ , Kapogiannis D (2017) Plasma extracellular vesicles enriched for neuronal origin: Aotential window into brain pathologic processes. Front Neurosci 11, 278. 28588440 10.3389/fnins.2017.00278 PMC5439289

[54] Folstein MF , Folstein SE , McHugh PR (1975) “Mini-mental state”. A practical method for grading the cognitive state of patients for the clinician. J Psychiatr Res 12, 189–198. 1202204 10.1016/0022-3956(75)90026-6

[55] Mohs RC , Knopman D , Petersen RC , Ferris SH , Ernesto C , Grundman M , Sano M , Bieliauskas L , Geldmacher D , Clark C , Thal LJ (1997) Development of cognitive instruments for use in clinical trials of antidementia drugs: Additions to the Alzheimer’s Disease Assessment Scale that broaden its scope. The Alzheimer’s Disease Cooperative Study. Alzheimer Dis Assoc Disord 11, S13–S21. 9236948

[56] Galasko D , Bennett D , Sano M , Ernesto C , Thomas R , Grundman M , Ferris S (1997) An inventory to assess activities of daily living for clinical trials in Alzheimer’s disease. The Alzheimer’s Disease Cooperative Study. Alzheimer Dis Assoc Disord 11, S33–S39. 9236950

[57] Knopman DS , Knap MJ , Gracon SI , Davis CS (1994) The Clinician Interview-Based Impression (CIBI): A clinician’s global change rating scale in Alzheimer’s disease. Neurology 44, 2315–2321. 7991118 10.1212/wnl.44.12.2315

[58] Mori Y , Tsuji M , Oguchi T , Kasuga K , Kimura A , Futamura A , Sugimoto A , Kasai H , Kuroda T , Yano S , Hieda S , Kiuchi Y , Ikeuchi T , Ono K (2021) Serum BDNF as a potential biomarker of Alzheimer’s disease: Verification through assessment of serum, cerebrospinal fluid, and medial temporal lobe atrophy. Front Neurol 12, 653267. 33967943 10.3389/fneur.2021.653267 PMC8102980

[59] Alvarez A , Aleixandre M , Linares C , Masliah E , Moessler H (2014) Apathy and APOE4 are associated with reduced BDNF levels in Alzheimer’s disease. J Alzheimers Dis 42, 1347–1355. 25024337 10.3233/JAD-140849 PMC4931817

[60] Alvarez A , Cacabelos R , Sanpedro C , García-Fantini M , Aleixandre M (2007) Serum TNF-alpha levels are increased and correlate negatively with free IGF-I in Alzheimer disease. Neurobiol Aging 28, 533–536. 16569464 10.1016/j.neurobiolaging.2006.02.012

[61] Pillai JA , Maxwell S , Bena J , Bekris LM , Rao SM , Chance M , Lamb BT , Leverenz JB ; Alzheimer’s Disease Neuroimaging Initiative (2019) Key inflammatory pathway activations in the MCI stage of Alzheimer’s disease. Ann Clin Transl Neurol 6, 1248–1262. 31353852 10.1002/acn3.50827 PMC6649519

[62] He M , Sun L , Cao W , Yin C , Sun W , Liu P , Tan L , Xu Z , Zhao W (2020) Association between plasma exosome neurogranin and brain structure in patients with Alzheimer’s disease: A protocol study. . BMJ Open 10, e036990. 10.1136/bmjopen-2020-036990 PMC7430441 32801201

[63] Alvarez XA , Sampedro C , Cacabelos R , Linares C , Aleixandre M , García-Fantini M , Moessler H (2009) Reduced TNF-α and increased IGF-I levels in the serum of Alzheimer’s disease patients treated with the neurotrophic agent cerebrolysin. Int J Neuropsychopharmacol 12, 867–872. 19531281 10.1017/S1461145709990101

[64] Alvarez XA , Alvarez I , Iglesias O , Crespo I , Figueroa J , Aleixandre M , Linares C , Granizo E , Garcia-Fantini M , Marey J , Masliah E , Winter S , Muresanu D , Moessler H (2016) Synergistic increase of serum BDNF in Alzheimer patients treated with cerebrolysin and donepezil: Association with cognitive improvement in ApoE4 cases. Int J Neuropsychopharmacol 19, pyw024. 27207906 10.1093/ijnp/pyw024 PMC4926802

